# Computed tomographic diaphragmatic thickness: a promising method for the evaluation of diaphragmatic muscle in cardiopulmonary diseased cats

**DOI:** 10.3389/fvets.2023.1247531

**Published:** 2023-12-14

**Authors:** Phasamon Saisawart, Somchin Sutthigran, Hathaiphat Suksangvoravong, Chutimon Thanaboonnipat, Sukullaya Ritthikulprasert, Kittipong Tachampa, Nan Choisunirachon

**Affiliations:** ^1^Department of Surgery, Faculty of Veterinary Science, Chulalongkorn University, Bangkok, Thailand; ^2^Department of Medicine, Faculty of Veterinary Science, Chulalongkorn University, Bangkok, Thailand; ^3^Department of Physiology, Faculty of Veterinary Science, Chulalongkorn University, Bangkok, Thailand

**Keywords:** cardiorespiratory, computed tomography, diaphragm, feline, measurement

## Abstract

Diaphragmatic dysfunction (DD) is defined as a weakening of the diaphragmatic muscle and can be an undetected cause of dyspnea. The objectives of this study were to explore the appropriate diaphragmatic location, measure diaphragmatic thickness (DT), evaluate the effect of intrinsic factors on DT, and compare DT between healthy and diseased cats, using 33 healthy cats and 15 diseased cats. A retrospective, analytical, case–control study using thoraco-abdominal feline computed tomography (CT) was performed. Two radiologists independently reviewed all images to verify inter- and intra-observer reliabilities and the best position for measuring DT. The effects of sex, age, and body weight were also studied, and cutoff values for detecting DT abnormalities were established. The results showed that the appropriate location for DT measurement was at the ventral border of the cranial endplate of the first lumbar vertebral body (L1) due to its highest intra- and inter-observer reliabilities. At this location, a significant difference in DT between the right and left hemidiaphragms (*p* = 0.01) was observed. Only sex had an impact on DT values. Interestingly, the DTs of cardiorespiratory-affected cats, both on the right and left sides, were significantly thinner than those of healthy cats. In conclusion, CT imaging is a reliable imaging method for determining diaphragmatic muscular atrophy. The ventral border of the cranial endplate of L1 is recommended for measuring the DT, and sex was the only factor affecting the DT measurement.

## 1 Introduction

Diaphragmatic dysfunction (DD) is a condition characterized by diaphragmatic muscle strength loss, which can be unilateral or bilateral. DD is an under-recognized cause of respiratory impairment, dyspnea, cyanosis, and death. It can be caused by several diseases involving the central nervous system, phrenic nerve, neuromuscular junction, and respiratory muscles (1, 2). In addition, conditions such as congestive heart failure (3), lung (4), pleural space (5), airway (6), and chronic kidney (7) diseases can affect diaphragmatic function. These diseases impair diaphragmatic function by increasing respiratory resistance and breathing workload. Subsequently, they reduce diaphragmatic movement and cause muscular atrophy.

Diaphragmatic dysfunction can be evaluated using clinical and/or diagnostic imaging parameters. Diagnostic imaging techniques can display anatomical features, such as diaphragmatic thickness (DT), diaphragmatic contraction (8), and movement (9, 10). Several imaging modalities have been utilized to determine diaphragmatic function, including radiography (11), fluoroscopy (11), ultrasonography (US) (8), magnetic resonance imaging (MRI) (11), and computed tomography (11, 12). However, each technique has its limitations, such as exposure to ionizing radiation from radiography, fluoroscopy, and CT, and the limited availability of MRI machines. Although the US is widely available, acts as a real-time, radiation-free technique, and provides high degrees of sensitivity and specificity, it is operator-dependent (11, 13).

Despite exposure to x-radiation, CT offers superior anatomical information about the lungs and airways. Recently, CT has gained popularity because it can be utilized as a diagnostic tool for respiratory illnesses in humans affected by COVID-19 (12, 14), chronic obstructive disorder (15), asthma (16), and DD (1, 15, 17, 18). Additionally, DT in patients with DD due to mechanical ventilation (17), chronic obstructive pulmonary disease (15), and diaphragmatic paralysis (1) can be evaluated using CT images.

A limited number of studies have demonstrated the application of diaphragmatic parameters, particularly diaphragmatic excursion, utilizing ultrasonographic methods in veterinary clinical practice. These include distinguishing between normal and paralyzed diaphragmatic motion (19), assessing diaphragmatic dysfunction in canines with cervical spinal disorders (20), and detecting diaphragmatic paralysis in various animal models, including a llama (21), cats (22), and a pony (23). Although DT is a reliable parameter for evaluating DD in humans, no information regarding computed tomographic DT measurements in companion animals is available. This study aimed to explore an appropriate diaphragmatic location to measure DT, to evaluate the effect of intrinsic factors such as age, body weight (BW), and sex on DT value, and to compare DT between healthy and diseased cats using CT images. We hypothesized that DT measurement is influenced by the location of the diaphragm and that DTs are different between healthy and diseased cats.

## 2 Materials and methods

### 2.1 Animals

This was a retrospective, analytical, case–control study. All the included retrospective data were approved by the Small Animal Hospital Committee of the Faculty of Chulalongkorn University (approval no. S.392/2565). All data from cats that underwent a thoraco-abdominal CT post-contrast protocol were included in the study. Clinical information such as age, BW, sex, neuter status, and previous history were obtained from digital medical records through the hospital information system. The patients were subsequently divided into two groups: healthy or diseased cats. The inclusion criteria for each group were as follows: Gr. I included CT images of cats that were clinically confirmed to be healthy and were acquired for other purposes such as intrathoracic structure studies (24), whereas Gr. II included CT images of cats with pulmonary parenchymal abnormalities, including interstitial or alveolar lung pattern, pleural effusion, and cardiovascular diseases. Patients were excluded if they had a history of diseases that could interfere with the diaphragmatic structure or neuromuscular function, such as diaphragmatic rupture, cervical intervertebral disease, previous diaphragmatic and cervical surgery, rib fractures, and intra-abdominal abnormalities inducing diaphragmatic compression, such as intra-abdominal organomegaly, pregnancy, or peritoneal effusion. In addition, CT images of inadequate quality due to the incomplete field of view and motion artifacts were excluded.

### 2.2 CT measurement protocol

From August 2018 to February 2023, CT images were collected using a 64-slice helical CT scanner (Optima CT660, GE Healthcare, Tokyo, Japan) under general anesthesia, performed by anesthesiologists. All cats were intubated, and anesthesia was maintained through a ventilator machine (SV-2000, SOARMED, Taiwan). CT images (512 × 512 matrix size, 0.625–1.25 mm slice thickness) were acquired within 10 min. Subsequently, CT images were retrieved from the Picture Archiving and Communication System in Digital and Communications in Medicine (DICOM) format and re-analyzed at a non-CT unit workstation with a 2,560 × 1,440-pixel monitor using DICOM viewer software (OsiriX, Geneva, Switzerland) at a window width of 350 Hounsfield units (HUs) and a window level of 40 HU by two radiologists with 3 years (P.S.) and 20 years (N.C.) of experience.

**Figure 1:**
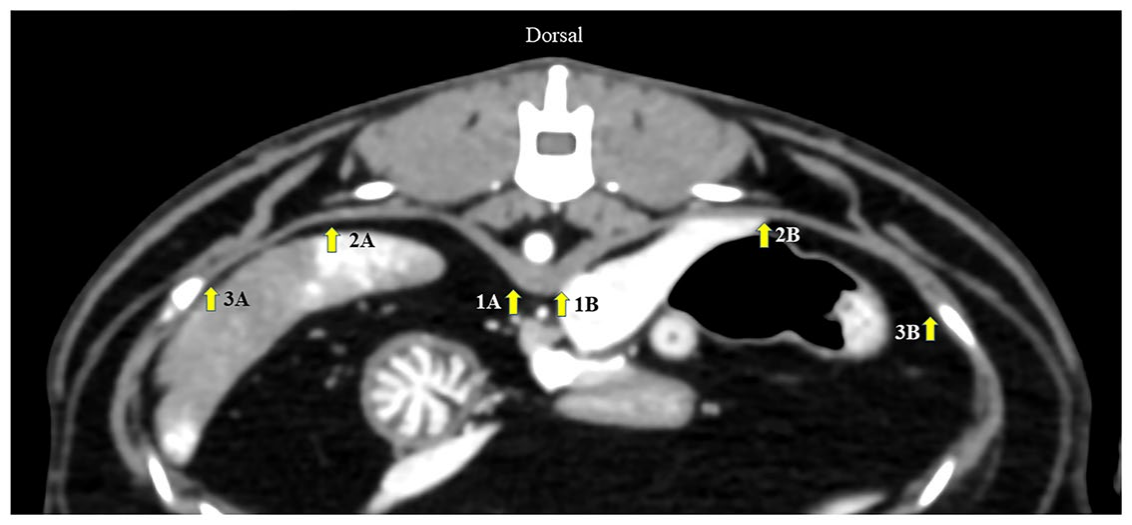
Axial computed tomography image demonstrating diaphragmatic thickness measurement from three different points: (1) at the level of the ventral border of the cranial endplate of L1, (2) the paramedian of each cupula, and (3) the most lateral of the costal part of the diaphragm. A represents the left diaphragm, and B represents the right diaphragm.

First, an appropriately diaphragmatic location for DT measurement was tested in Gr. I. Multiplanar reconstruction was performed to reveal the crura of each hemidiaphragm at the cranial endplate of the first lumbar vertebrae (L1) on the axial plane. The DTs of each hemidiaphragm were measured using a digital caliper in three different areas of the dorsal diaphragmatic location (20): (i) at the ventral border of L1; (ii) at the paramedian area of each cupula; and (iii) at the most lateral part attached to the coastal area (Figure 1). Then, inter- and intra-observer reliabilities were tested. To assess intra-observer reliability, CT images were examined twice by the same operator, 1 week apart. To test inter-observer reliability, CT images were measured by two experienced operators in a randomized order for each measurement. An appropriate diaphragmatic location for DT measurement was selected according to the highest intra- and inter-observer reliabilities. The same measurement technique was used to evaluate the DT in Gr. II.

**Figure 2:**
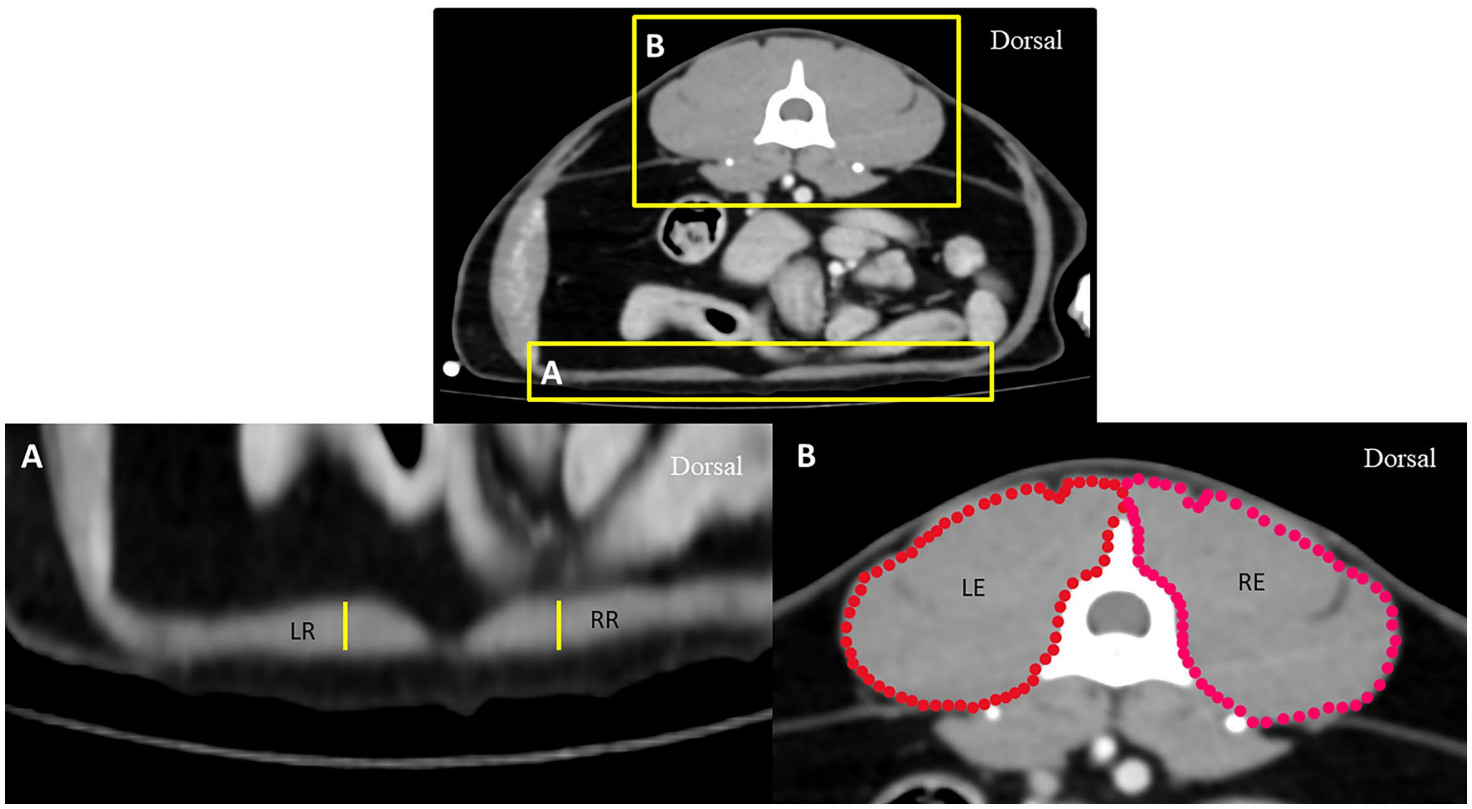
Axial computed tomography demonstrating measurement methods for the thickness of the rectus abdominis muscle **(A)** and the surface of epaxial muscle **(B)** at the level of the L4 vertebra: RR represents the thickness of the rectus abdominis muscle on the right side, LR represents the thickness of the rectus abdominis muscle on the left side, RE represents the surface of the right epaxial muscle, and LE represents the surface of the left epaxial muscle.

To eliminate the effect of body size variation on DT, the aortic diameter (Ao) was measured at the ventral border of the cranial endplate of L1 and compared between the groups. In addition, to eliminate the effect of general muscular volume between groups, the right and left epaxial muscle surfaces, including the rectus abdominis muscle thicknesses at the mid-abdomen indicated by the level of the cranial part of the fourth lumbar vertebrae (L4), were also measured and compared between groups (Figure 2). All parameters were measured thrice and averaged.

### 2.3 Statistical analysis

Data were analyzed using Prism8 (GraphPad Software, CA, United States). The normality of each data set was analyzed using the Shapiro–Wilk test. All clinical data on enrolled cats were described as descriptive data. Inter-observer- and intra-observer reliabilities were evaluated using intraclass correlation coefficient scores with 95% confidence intervals (CIs). The effect of age, BW, and sex on right and left DTs was assessed using Pearson’s correlation analysis. A Student’s t-test was used for comparisons between groups. The receiver operating characteristic (ROC) curve for Gr. I and Gr. II was used to calculate the DT cutoff values. The 95% confidence interval (CI) and area under the ROC curve (AUC) were calculated. Statistical significance was defined as a *p*-value of <0.05.

## 3 Results

### 3.1 Clinical demographic data

In this study, 48 cats met the inclusion criteria and were then divided into 2 groups: 33 cats in Gr. I and 15 cats in Gr. II. Patients’ demographic characteristics are shown in Table 1. Cats in Gr. II were significantly older than those in Gr. I. BWs were similar between the two groups. In Gr. I, male cats were significantly heavier than female cats (*p* = 0.009). In addition, female cats were significantly older than male cats in Gr. II (*p* = 0.02). There were Domestic Shorthair (*n* = 23), American Shorthair (*n* = 3), Persian (*n* = 5), and one cat from each of the following breeds of Scottish Fold and Khoa Manee in Gr. I, whereas cats in Gr. II were all Domestic Shorthair (*n* = 15). Cats in Gr. II were affected by lung nodules (*n* = 6), pleural effusion (*n* = 4), interstitial diseases (*n* = 2), congestive heart failure (*n* = 1), intrathoracic mass (*n* = 1), and pneumonia (*n* = 1).

**Table 1:** Clinical demographic information among 33 healthy cats and 15 diseased cats.

	Healthy (Gr. I)	Diseased (Gr. II)
Number	33	15
Sex		
Male	18	2
Female	15	13
Age (month): overall	52.50 ± 50.90*	86.77 ± 49.03*
Male	49.50 ± 53.15	48.00 ± 16.97*
Female	55.42 ± 49.91	93.82 ± 50.00*
BW (kg): overall	3.56 ± 1.18	3.54 ± 0.81
Male	4.16 ± 1.04**	3.90 ± 1.41
Female	3.10 ± 1.09**	3.49 ± 0.77

Data were expressed as mean ± SD. * Significant difference of age between Gr. I and Gr. II was made by unpaired *t*-test; *p* = 0.02. ** Significant difference of BW between male and female cats in Gr. I was made by unpaired *t*-test; *p* = 0.009.

### 3.2 Diaphragmatic location for DT measurement

The DT values among three locations of each hemidiaphragm in Gr. I, including the inter- and intra-observer reliabilities, are shown in Table 2. The DT at the ventral border of the cranial endplate of L1 had the highest inter- and intra-observer reliabilities. Therefore, this location was selected to evaluate DT for the remainder of the experiment.

**Table 2:** DT values, inter-, and intra-observer reliability for the measurements of diaphragmatic thickness for each location.

	DT value	Intraclass correlation coefficient	DT value	Intraclass correlation coefficient
Inter-observer reliability				
The ventral border of vertebrae	2.13 ± 0.92	0.849 (0.742 – 0.920)	2.45 ± 0.93	0.862 (0.747 – 0.927)
The paramedian of each cupula	1.71 ± 0.41	0.526 (0.244 – 0.727)	1.85 ± 0.61	0.775 (0.602 – 0.878)
The most lateral of the costal part of the diaphragm	1.79 ± 0.60	0.627 (0.380 – 0.790)	1.77 ± 0.48	0.755 (0.570 – 0.867)
Intra-observer reliability				
The ventral border of vertebrae	2.02 ± 0.98	0.963 (0.925 – 0.981)	2.34 ± 1.21	0.862 (0.747 – 0.927)
The paramedian of each cupula	2.57 ± 1.61	0.755 (0.397 – 0.965)	2.69 ± 1.42	0.775 (0.227 – 0.950)
The most lateral of the costal part of the diaphragm	2.73 ± 0.74	0.840 (0.397 – 0.965)	2.82 ± 1.09	0.846 (0.414 – 0.967)

Data were expressed as mean ± SD and 95% confidence intervals.

### 3.3 Comparison of DT values between healthy and diseased cats

**Figure 3:**
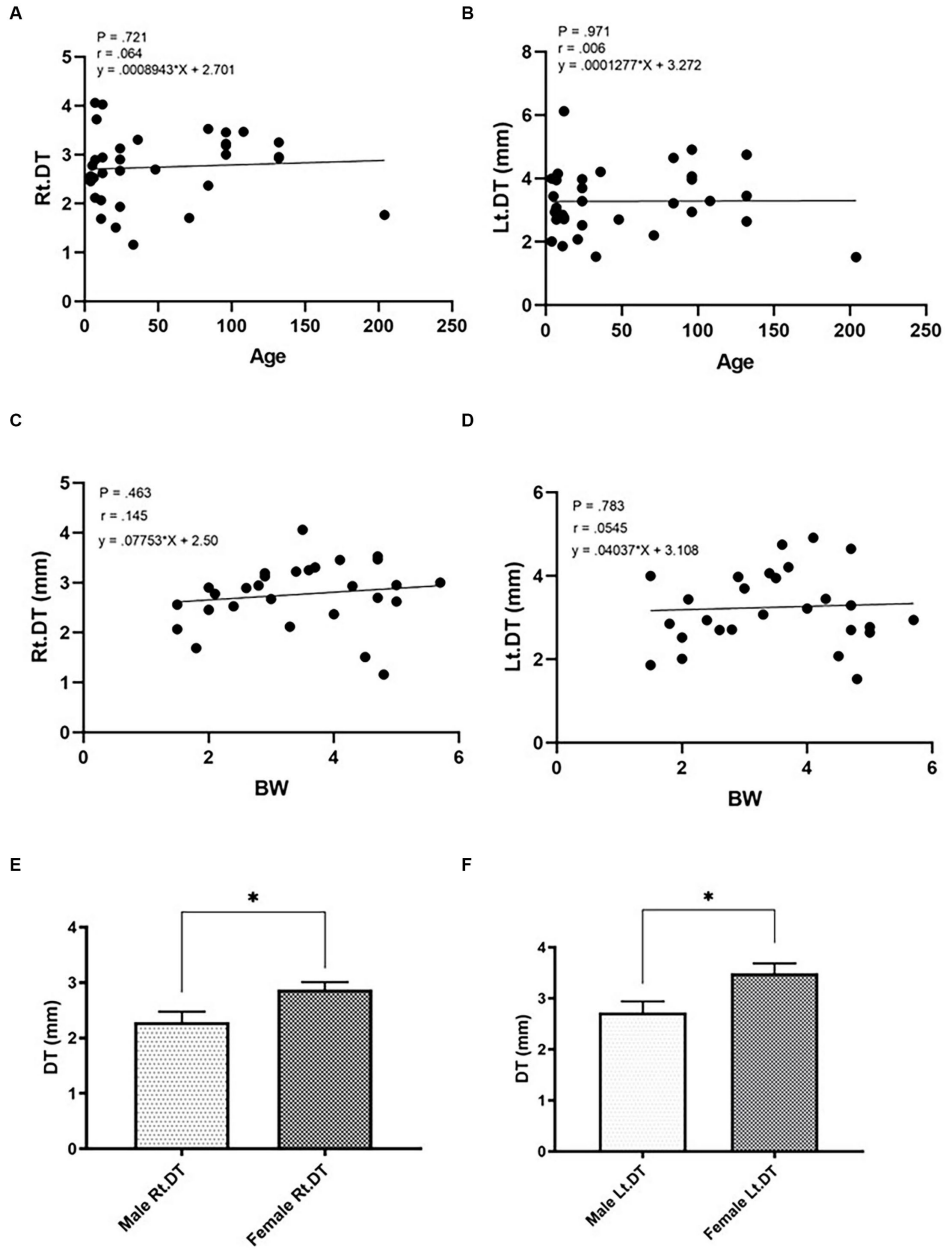
Correlations between the diaphragmatic thickness (DT) and intrinsic factors of cats such as age (**A,B**; **A** = right hemidiaphragm, **B** = left hemidiaphragm), BW (**C,D**; **C** = right hemidiaphragm, **D** = left hemidiaphragm), and sex (**E,F**; **E** = male, **F** = female) of healthy cats. *Significant difference; *p* < 0.05.

The DT values of cats in Gr. I and Gr. II are listed in Table 3. In Gr. I, the DT of the left hemidiaphragm at the level of L1 was significantly higher than that on the right side (*p* = 0.014), whereas the DTs of Gr. II were similar on each side. The effect of clinical demographic data on the DTs of cats in Gr. I is shown in Figure 3. Age (*r* = 0.064, *p* = 0.721; *r* = 0.006, 141 *p* = 0.971, for the right and left hemidiaphragms, respectively) and BW (*r* = 0.145, *p* = 0.463; *r* = 142 0.054, *p* = 0.783, for the right and the left hemidiaphragm, respectively) were not significantly correlated with the DTs. However, the DTs of the right and the left hemidiaphragm of females were higher than those of males (*p* = 0.013 and 0.014 for the right and left hemidiaphragm, respectively).

**Table 3:** Diaphragmatic thickness and other parameters between healthy and diseased cats.

Parameters	Healthy cats (Gr. I)	Diseased cats (Gr. II)
DT (mm)		
Right DT	2.74 ± 0.65*, ****	1.74 ± 0.76****
Left DT	3.25 ± 0.87*, ****	1.64 ± 0.47****
Aortic diameters (mm)	3.99 ± 0.93	4.26 ± 1.44
Surface of epaxial muscle (cm^2^)		
Right epaxial muscle	5.23 ± 1.49	4.99 ± 1.89
Left epaxial muscle	5.26 ± 1.53	5.05 ± 1.99
Rectus abdominis thickness (mm)		
Right side	2.83 ± 0.63	3.61 ± 1.17**
Left side	2.93 ± 0.56	3.65 ± 1.22**

Data were expressed as mean ± SD. * Significant difference of DT value between right and left hemidiaphragm in Gr. I was made by unpaired *t*-test; *p* = 0.014. ** Significant difference in rectus abdominis thickness between Gr. I and Gr. II was made by unpaired *t*-test; *p* = 0.005 for the right and *p* = 0.009 for the left hemidiaphragm, respectively. **** Significant difference of DT values between Gr. I and Gr. II was made by unpaired *t*-test; *p* = 0.0001 for both right and left hemidiaphragm.

Considering both the right and left hemidiaphragm, DTs in Gr. I were significantly higher than those in Gr. II (*p* < 0.0001 and *p* < 0.0001 for the right and left hemidiaphragm, respectively). The Ao and surface of the epaxial muscles were not significantly different between groups. However, the rectus abdominis thickness in Gr. II was significantly higher than that of Gr. I (*p* = 0.005 and *p* = 0.009 for the right and left hemidiaphragms, respectively) (Table 3).

**Figure 4:**
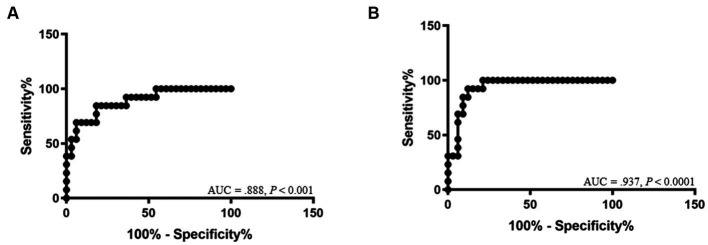
Receiver-operating characteristic curves show the area under the curve (AUC) and *p-*value of significance for the diagnosis of diaphragmatic dysfunction of the right hemidiaphragm **(A)** and left hemidiaphragm **(B)**.

The ROC curve for DT is shown in Figure 4. The cutoff values of DT between Gr. I and Gr. II were 2.04 mm for the right (AUC: 0.888, 95% CI: [0.784–0.992]; *p* < 0.001, with a sensitivity of 84.6% and a specificity of 81.8%), and 2.06 mm for the left hemidiaphragm (AUC: 0.937, 95% CI: [0.869–1.00]; *p* < 0.0001, with a sensitivity of 92.3% and a specificity of 87.9%), respectively.

## 4 Discussion

The diaphragm is the principal muscle involved in respiration. Diseases that reduce diaphragm thickness can lead to DD. Subsequently, it affects respiration, induces hypoxia, and causes cyanosis and death (4, 25, 26). Although a thin DT is not a direct diaphragmatic abnormality, it reflects a weakening of the muscular function of the diaphragm. This study is the first to investigate computed tomographic DT values in healthy and diseased cats. In the healthy cat group, DT values were 2.74 ± 0.65 mm and 3.25 ± 0.87 mm for the right and left hemidiaphragms, respectively. For the diseased group, DT values were 1.74 ± 0.76 mm and 1.64 ± 0.47 mm for the right and left hemidiaphragm, respectively. In addition, we established cutoff values of 2.04 mm for the right hemidiaphragm (sensitivity 84.6%, specificity 81.8%) and 2.06 mm for the left hemidiaphragm (sensitivity 92.3%, specificity 87.9%). These results can be used as references in future.

To the best of our knowledge, this is the first study to explore the optimal location for DT measurement using CT in animals. Our study showed that the ventral border of the L1 cranial endplate on the axial plane was the most reliable location because of its high intra- and inter-observer reliabilities, which is consistent with a previous study in humans (27). Determining the outline of the diaphragmatic muscle is difficult at the paramedian diaphragmatic area of each cupula and at the most lateral diaphragmatic part attached to the costal bone because it is thin and its attenuation is equal to the liver on the right side and the spleen on the left side, even on the post-contrast enhancement phase. Which explains the lower inter- and intra-observer reliabilities at these locations. Furthermore, the left hemidiaphragm was thicker than the right hemidiaphragm at the ventral border of the L1 cranial endplate in our study, which is in contrast to several studies in humans in which the right hemidiaphragm was reported to be thicker than the left (27). According to the normal anatomy of the cat, the diaphragmatic muscles in the lumbar region originate in pairs from the ventral aspects of the first three or four lumbar vertebrae and fan out to join the central tendon in the dorsal regions. The right crus is considerably larger than the left at the ventral border of L3 or L4. Because the right crus originates more caudally than the left crus, it becomes thinner earlier, which may explain this finding at the level of L1 (28).

Diseased cats were older than healthy cats, which was expected, as the incidence of cardiorespiratory diseases is higher in aged cats. Age or BW did not affect DT in healthy cats. However, sex was a significant factor influencing DT. Most human studies found a discrepancy in this information in that the DTs of male patients were thicker than those of females (8, 13, 29), while another study claimed that there was no statistical difference in DT between sexes (30). Martin et al. have reported that low levels of testosterone could impair skeletal muscle performance, causing it to become thinner and weaker, especially in the diaphragm (31). Healthy males normally have higher testosterone levels than females (32, 33), which may be the main factor causing a thicker DT in humans. However, the feline DTs between sexes were contrasted in our study, as the DT of females was thicker than that of males. This may be due to species-specific conditions. Therefore, a prospective study to evaluate the DT in different sexes, comparing the relative hormonal factors, should be conducted to provide more information.

Several parameters, such as BW, Ao, epaxial muscle surface, and rectus abdominis muscle thickness, were compared to minimize the effect of chronic illness on the different general muscular volumes between groups. The results showed no statistical differences in BW, Ao, or epaxial muscle surface. This may imply that the body sizes observed through BW, Ao, and the general muscular volume observed through the epaxial muscle surface in both groups were similar. Therefore, the DT of healthy and diseased cats differs in concurrence with intrathoracic diseases. To observe the general muscle volume through both the epaxial muscle surface and the thickness of the accessory respiratory muscles in the abdomen through the rectus abdominis, the mid-abdominal area at the cranial part of L4 was selected as the location for evaluation because of its simple applicability. Interestingly, the muscles of diseased cats had significantly greater rectus abdominis thickness than those of healthy cats. This might be due to an accessory respiratory muscle such as the rectus abdominis, which has a greater workload to compensate for breathing during diaphragmatic weakness (34).

This study had some limitations. First, the CT protocol was not standardized because this was a retrospective study. Second, the degree of crus atrophy may have been affected by the timing of the CT scans after the onset of DD, and we were unable to determine the degree of chronicity of these disorders. Third, despite the obvious difference in DTs between groups, DTs can be different between the respiration phases. The DT will be thicker during inspiration and thinner during expiration (35). The current study, using CT images, could not compare DTs between the respiratory phases due to being a retrospective model. Therefore, the influencing effect of the respiratory phase on the normal and disease-affected diaphragm should be further investigated using respiratory-gated CT images or US to see how the breathing cycle affects the diaphragm in both healthy cats and those with breathing problems for a more reliable result.

In conclusion, a reliable location for measuring DT in cats is the ventral border of the cranial endplate of L1, owing to its high intra- and inter-observer reliabilities. Age and BW did not affect DT value in healthy cats; however, sex played a significant role in determining DT value. A cutoff value of 2.04 and 2.06 mm for the right and left hemidiaphragms, respectively, with high sensitivity and specificity, can be used as a guideline to predict DD in cats without interference from chronic illness-induced muscular atrophy.

## Data availability statement

The raw data supporting the conclusions of this article will be made available by the authors, without undue reservation.

## Author contributions

PS and NC performed the concept/design, data analysis/interpretation, drafting of the manuscript, and critical revision of the manuscript. SS performed data analysis/interpretation, critical revision of the manuscript, and approved the manuscript. HS, SR, KT, and CT critically revised the manuscript and approved the manuscript. All authors contributed to the article and approved the submitted version.

## References

[1] Sukkasem W Moftah SG Kicska G Godwin JD Pipavath S Stern E. Crus atrophy: accuracy of computed tomography in diagnosis of diaphragmatic paralysis. J Thorac Imaging. (2017) 32:6. doi: 10.1097/RTI.0000000000000276 28549021

[2] McCool FD Tzelepis GE. Dysfunction of the diaphragm. N Engl J Med. (2012) 366:932–42. doi: 10.1056/NEJMra1007236 22397655

[3] Cross TJ Kim CH Johnson BD Lalande S. The interactions between respiratory and cardiovascular systems in systolic heart failure. J Appl Physiol (1985). (2020) 128:214–24. doi: 10.1152/japplphysiol.00113.2019 31774354

[4] Ricoy J Rodríguez-Núñez N Álvarez-Dobaño JM Toubes ME Riveiro V Valdés L. Diaphragmatic dysfunction. Pulmonology. (2019) 25:223–35. doi: 10.1016/j.pulmoe.2018.10.008 30509855

[5] Aguilera Garcia Y Palkar A Koenig SJ Narasimhan M Mayo PH. Assessment of diaphragm function and pleural pressures during thoracentesis. Chest. (2020) 157:205–11. doi: 10.1016/j.chest.2019.07.019 31398347

[6] Corbellini C Boussuges A Villafañe JH Zocchi L. Diaphragmatic mobility loss in subjects with moderate to very severe COPD may improve after in-patient pulmonary rehabilitation. Respir Care. (2018) 63:1271–80. doi: 10.4187/respcare.06101 30065081

[7] Figueiredo PH Lima MM Costa HS Gomes RT Neves CD Oliveira ES. The role of the inspiratory muscle weakness in functional capacity in hemodialysis patients. PLoS One. (2017) 12:e0173159. doi: 10.1371/journal.pone.0173159 28278163 PMC5344350

[8] Boussuges A Rives S Finance J Chaumet G Vallée N Risso JJ. Ultrasound assessment of diaphragm thickness and thickening: reference values and limits of normality when in a seated position. Front Med (Lausanne). (2021) 8:742703. doi: 10.3389/fmed.2021.742703 34778304 PMC8579005

[9] Boussuges A Finance J Chaumet G Brégeon F. Diaphragmatic motion recorded by m-mode ultrasonography: limits of normality. ERJ Open Res. (2021) 7:00714–2020. doi: 10.1183/23120541.00714-2020 33778044 PMC7983192

[10] Boussuges A Rives S Finance J Brégeon F. Assessment of diaphragmatic function by ultrasonography: current approach and perspectives. World J Clin Cases. (2020) 8:2408–24. doi: 10.12998/wjcc.v8.i12.2408 32607319 PMC7322428

[11] Laghi FA Saad M Shaikh H. Ultrasound and non-ultrasound imaging techniques in the assessment of diaphragmatic dysfunction. BMC Pulm Med. (2021) 21:85. doi: 10.1186/s12890-021-01441-6 33722215 PMC7958108

[12] Luo N Zhang H Zhou Y Kong Z Sun W Huang N. Utility of chest CT in diagnosis of COVID-19 pneumonia. Diagn Interv Radiol. (2020) 26:437–42. doi: 10.5152/dir.2020.20144 32490829 PMC7490028

[13] Boon AJ Sekiguchi H Harper CJ Strommen JA Ghahfarokhi LS Watson JC. Sensitivity and specificity of diagnostic ultrasound in the diagnosis of phrenic neuropathy. Neurology. (2014) 83:1264–70. doi: 10.1212/WNL.0000000000000841 25165390 PMC4180486

[14] Aljondi R Alghamdi S. Diagnostic value of imaging modalities for COVID-19: scoping review. J Med Internet Res. (2020) 22:e19673. doi: 10.2196/19673 32716893 PMC7468642

[15] Donovan AA Johnston G Moore M Jensen D Benedetti A Coxson HO. Diaphragm morphology assessed by computed tomography in chronic obstructive pulmonary disease. Ann Am Thorac Soc. (2021) 18:955–62. doi: 10.1513/AnnalsATS.202007-865OC 33321048

[16] Walker C Gupta S Hartley R Brightling CE. Computed tomography scans in severe asthma: utility and clinical implications. Curr Opin Pulm Med. (2012) 18:42–7. doi: 10.1097/MCP.0b013e32834db255 22112997 PMC3387553

[17] Lee GD Kim HC Yoo JW Lee SJ Cho YJ Bae K. Computed tomography confirms a reduction in diaphragm thickness in mechanically ventilated patients. J Crit Care. (2016) 33:47–50. doi: 10.1016/j.jcrc.2016.02.013 26979912

[18] Ni Y-N Xu H Li W-J Sun J-K Liang B-M Liang Z-A. Could the loss of diaphragm thickness measured by computer tomography predict the rate of reintubation? J Thorac Disease. (2020) 12:581–91. doi: 10.21037/jtd.2019.12.125 32274124 PMC7138965

[19] Choi M Lee N Kim A Keh S Lee J Kim H. Evaluation of diaphragmatic motion in normal and diaphragmatic paralyzed dogs using m-mode ultrasonography. Vet Radiol Ultrasound. (2014) 55:102–8. doi: 10.1111/vru.12126 24267008

[20] Drury BL Brinkman EL Gambino JM Lee AM Wills RW Beasley MJ. Diaphragmatic dysfunction in dogs with cervical spinal disorders before and after surgery using fluoroscopy, motion-mode ultrasound and radiography was not different than a group of control dogs. Vet Radiol Ultrasound. (2020) 61:353–63. doi: 10.1111/vru.12833 31899935

[21] Bedenice D Mazan MR Kuehn H Hoffman AM. Diaphragmatic paralysis due to phrenic nerve degeneration in a llama. J Vet Intern Med. (2002) 16:603–6. doi: 10.1892/0891-6640(2002)016<0603:dpdtpn>2.3.co;2 12322714

[22] Vignoli M Toniato M Rossi F Terragni R Manzini M Franchi A. Transient post-traumatic hemidiaphragmatic paralysis in two cats. J Small Anim Pract. (2002) 43:312–6. doi: 10.1111/j.1748-5827.2002.tb00080.x 12137153

[23] Amory H Lomba F Lekeux PM Solal AN Jauniaux TP Desmecht DJ. Bilateral diaphragmatic paralysis in a pony. J Am Vet Med Assoc. (1994) 205:587–91. 7961096

[24] Thammasiri N Thanaboonnipat C Choisunirachon N Darawiroj D. Multi-factorial considerations for intra-thoracic lymph node evaluations of healthy cats on computed tomographic images. BMC Vet Res. (2021) 17:59. doi: 10.1186/s12917-021-02771-7 33509167 PMC7844987

[25] Dubé BP Dres M. Diaphragm dysfunction: diagnostic approaches and management strategies. J Clin Med. (2016) 5:13. doi: 10.3390/jcm5120113 27929389 PMC5184786

[26] Mirabile VS Shebl E Sankari A Burn B. Respiratory failure. In: StatPearls. Treasure Island (FL): StatPearls Publishing (2023) Available at: https://www.ncbi.nlm.nih.gov/books/NBK526127/. 30252383

[27] Ufuk F Çakmak P Sagtas E Herek D Arslan M Yagci B. The diaphragm thickness measurements on computed tomography: intra- and inter-observer reliability. Istanb Med J. (2019) 20:101–6. doi: 10.4274/imj.65471

[28] Hudson LC Hamilton WP. Respiratory system In: Smith B, editor. Atlas of feline anatomy for veterinarian. Jackson, WY: Teton Newmedia (2010). 144.

[29] Scarlata S Mancini D Laudisio A Benigni A Antonelli IR. Reproducibility and clinical correlates of supine diaphragmatic motion measured by m-mode ultrasonography in healthy volunteers. Respiration. (2018) 96:259–66. doi: 10.1159/000489229 30114702

[30] Oguri M Okanishi T Ikeguchi T Ogo K Kanai S Maegaki Y. Influence of gender on diaphragm thickness using a method for determining intima media thickness in healthy young adults. BMC Med Imaging. (2022) 22:26. doi: 10.1186/s12880-022-00748-y 35148697 PMC8840635

[31] Martins G Verdeal JCR Tostes H da Silva ARO Tessarollo B Rocha NN. Testosterone therapy and diaphragm performance in a male patient with COVID-19: a case report. Diagnostics. (2022) 12:535. doi: 10.3390/diagnostics12020535 35204624 PMC8871258

[32] Clark RV Wald JA Swerdloff RS Wang C Wu FCW Bowers LD. Large divergence in testosterone concentrations between men and women: frame of reference for elite athletes in sex-specific competition in sports, a narrative review. Clin Endocrinol. (2019) 90:15–22. doi: 10.1111/cen.13840 30136295

[33] van Anders SM Steiger J Goldey KL. Effects of gendered behavior on testosterone in women and men. Proc Natl Acad Sci. (2015) 112:13805–10. doi: 10.1073/pnas.1509591112 26504229 PMC4653185

[34] LoMauro A Aliverti A Perchiazzi G Frykholm P. Physiological changes and compensatory mechanisms by the action of respiratory muscles in a porcine model of phrenic nerve injury. J Appl Physiol. (2021) 130:813–26. doi: 10.1152/japplphysiol.00781.2020 33444121

[35] Hall JE. Pulmonary ventilation In: Hall JE, editor. Guyton and Hall textbook of medical physiology. Philadelphia, PA: Elsevier (2011). 465–6.

